# Mechanistic Systems Biology of High-Salinity Fermented Seafood: Multi-Omics Integration for Microbial Safety and Quality Prediction

**DOI:** 10.3390/biology15100772

**Published:** 2026-05-12

**Authors:** Mia Yang Ang, Chen Li, Heru Pramono, Teck Yew Low, Nur Azalina Suzianti Feisal, Guat Jah Wong, Siew Woh Choo

**Affiliations:** 1 Department of Biomedical Sciences, Jeffrey Cheah Sunway Medical School, Faculty of Medical and Life Sciences, Sunway University, Sunway City, Petaling Jaya 47500, Selangor, Malaysia; 2 Sunway Microbiome Centre, Faculty of Medical and Life Sciences, Sunway University, Sunway City, Petaling Jaya 47500, Selangor, Malaysia; 3 Zhejiang-Malaysia Joint Laboratory for Rare Medicinal Resources, Wenzhou-Kean University, 88 Daxue Road, Ouhai, Wenzhou 325060, China; 4 College of Science, Mathematics and Technology, Wenzhou-Kean University, 88 Daxue Road, Ouhai, Wenzhou 325060, China; 5 Department of Marine, Faculty of Fisheries and Marine, Universitas Airlangga, Surabaya 60115, East Java, Indonesia; 6 MSU Centre for Climate Resilience and Strategy (m-CREST), Management and Science University, University Drive, Off Persiaran Olahraga, Shah Alam 40100, Selangor, Malaysia; 7 Department of Diagnostics and Allied Health Science, Faculty of Health and Life Sciences, Management and Science University, University Drive, Off Persiaran Olahraga, Shah Alam 40100, Selangor, Malaysia; 8 International Frontier Interdisciplinary Research Institute (IFIRI), Wenzhou-Kean University, 88 Daxue Road, Ouhai, Wenzhou 325060, China; 9 Dorothy and George Hennings College of Science, Mathematics and Technology, Kean University, 1000 Morris Ave, Union, NJ 07083, USA

**Keywords:** multi-omics, systems biology, high-salinity fermentation, fermented seafood, predictive food safety

## Abstract

Foodborne diseases present a serious public health challenge, causing roughly 600 million illnesses and 420,000 deaths annually. A significant portion of this impact is felt in Asia, where traditional fermented and dry-salted seafood, such as *katsuobushi*, *budu*, and *peda*, are dietary staples. These products rely on diverse microbial communities that determine their final safety, flavor, texture, and shelf life. Historically, research has centered on lactic acid bacteria (LAB), yet the functional contributions of non-LAB halotolerant species, including genera like *Tetragenococcus*, *Staphylococcus*, and *Bacillus*, are functionally important in these high-salinity niches. This review evaluates the transition from basic taxonomic surveys to mechanistic multi-omics approaches, integrating genomics, transcriptomics, proteomics, and metabolomics to decode microbial functionality under selective environmental pressures. We discuss how genomic mining using platforms such as BAGEL4 and antiSMASH can uncover biosynthetic gene clusters and antimicrobial peptides, while CARD supports antimicrobial resistance monitoring. Transcriptomic analysis reveals microbial responses to osmotic stress, low water activity, and pH fluctuations, whereas proteomic profiling links gene expression to active enzymes, stress proteins, and functional biomarkers. Metabolomics captures the chemical outcomes of fermentation, including amino acids, volatile organic compounds, spoilage markers, and biogenic amines. By merging these high-dimensional datasets with artificial intelligence, researchers can move toward predictive modeling that distinguishes biological causation from simple correlation. This shift offers a strategy to improve the safety, consistency, and resilience of traditional high-salinity fermented seafood systems.

## Simple Summary

Traditional fermented and salted seafood products are important cultural and dietary foods, particularly in Asia. Their safety and quality depend on complex microbial communities that survive under strong environmental pressures, including high salt concentration, low water availability, pH changes, oxygen variation, and long fermentation periods. Earlier studies often focused on identifying which microorganisms are present, especially lactic acid bacteria. However, microbial presence alone does not explain how these organisms adapt, interact, produce desirable flavor compounds, or generate safety-related hazards such as biogenic amines. This review discusses how multi-omics approaches, including genomics, transcriptomics, proteomics, and metabolomics, can be integrated to understand microbial function in high-salinity fermented seafood. Rather than focusing mainly on databases and analytical tools, the review emphasizes the biological mechanisms linking microbial communities, environmental selection, enzyme activity, metabolite formation, and food safety outcomes. A conceptual framework based on histamine formation in *Tetragenococcus halophilus* is used to show how gene presence, gene expression, enzyme activity, and metabolite accumulation can be connected into a predictive safety model. This systems-level perspective can support earlier risk detection, improved process control, and more consistent production of safe, high-quality traditional fermented seafood.

## 1. Introduction: Food Safety and Microbial Ecology of High-Salinity Fermented Seafood

Food safety is a fundamental pillar of public health, directly impacting the reduction in foodborne illnesses globally [1]. The health burden associated with unsafe food remains severe, with approximately 600 million annual cases of foodborne disease resulting in 420,000 deaths [2]. This situation is particularly acute in Southeast Asia, where an estimated 150 million cases and 175,000 deaths occur every year [3]. Addressing these complexities requires a transition from descriptive monitoring toward a mechanistic understanding of how microbes function within traditional food systems.

### 1.1. The Microbial Landscape and Environmental Selection

Traditional fermented and dry-salted seafood products are dietary staples across Asia and parts of Europe [4,5]. These foods rely on complex microbial ecosystems governed by strong environmental selection pressures—such as osmotic stress, pH fluctuations, and oxygen availability—which define their final sensory attributes, safety, and shelf life [6]. Historically, research centered on lactic acid bacteria (LAB) because of their ability to inhibit pathogens, enhance flavor, and extend product stability [7,8]. However, recent data show that non-LAB organisms, including halotolerant and halophilic taxa such as *Tetragenococcus*, *Staphylococcus*, *Bacillus*, and halophilic archaea, play critical functional roles in these high-salinity niches [9].

### 1.2. Transitioning from Descriptive to Predictive Frameworks

**Figure 1:**
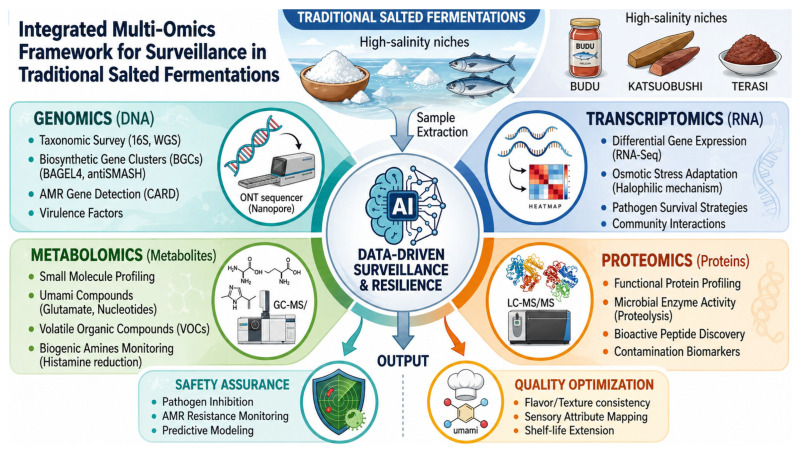
Integrated multi-omics framework for predictive surveillance in high-salinity fermented seafood. The workflow links sample collection with four omics layers: genomics for taxonomic profiling and antimicrobial resistance detection, transcriptomics for microbial stress responses, proteomics for functional enzyme profiling, and metabolomics for bioactive compounds and biogenic amines. Integrated analysis supports predictive safety monitoring, pathogen mitigation, sensory quality assessment, and shelf-life optimization.

While the rapid advancement of omics technologies now enables a high-resolution analysis of these microbial communities, moving beyond simple taxonomic surveys requires an integrated systems biology framework. As shown in Figure 1, this approach synthesizes data from genomics, transcriptomics, proteomics, and metabolomics to provide a foundation for AI-assisted predictive surveillance in traditional salted fermentations. By mapping these molecular layers, it becomes possible to distinguish between simple microbial presence and active metabolic causation, effectively bridging the gap between complex microbial data and actionable food safety risk management.

## 2. Ecological Diversity and Environmental Selection in High-Salinity Food Matrices

Fermented seafood products are produced in diverse physical forms—solids, liquids, and pastes—each of which establishes a unique set of selective conditions for microbial growth [10]. These matrices impose distinct environmental filters, primarily osmotic pressure, fluctuating pH, and varying oxygen availability, which collectively dictate the microbial succession and functional output of the ecosystem [10,11,12,13,14].

### 2.1. Diversity Across Physical Fermentation Niches

The physical structure of the food matrix plays a decisive role in shaping the microbial niche. Solid fermented seafood, such as Japanese *katsuobushi* (fermented bonito) [15] and Indonesian *peda (fermented mackerel)* [16], often selects for specialized aerobic and facultative anaerobic species capable of colonizing both surface and interior under limited water activity and oxygen gradients.

In contrast, liquid products, such as fish sauces, including Malaysian *budu* [17], Vietnamese *nước mắm* [18], Indonesian *kecap ikan* [19], and Thai *nam pla* [20] feature different salt diffusion and moisture profiles. Furthermore, paste-form products, such as Indonesian *terasi* [21], Thai *pla som* [22], and Malaysian *cincalok* [23] present unique challenges regarding oxygen exposure and protein availability. Across these matrices, the duration of fermentation and the protein-to-salt ratio significantly influence how microbial communities evolve and which metabolites they produce.

### 2.2. Osmotic Stress and Microbial Adaptation Strategies

The extreme salinity inherent to liquid and paste matrices, often exceeding 20% NaCl, acts as a strong selective filter. This environment favors halotolerant bacteria such as *Tetragenococcus*, *Staphylococcus*, and *Bacillus*, alongside halophilic archaea that thrive under extreme salt conditions [24]. To survive, these microbes employ specialized ‘salt-in’ or ‘salt-out’ strategies to maintain cellular turgor against the surrounding brine [25].

These adaptation patterns are evident across several regional systems. For example, metabarcoding has highlighted specific microbiota in Thai *Pla Ra* and Laotian *Pla daek* [11], while proteomics-based approaches have been used to characterize Cambodian *Prohok* [26] and South Korean *Jeotgal* [12].

### 2.3. A Unified Framework for Functional Succession

Metabolomic tools, particularly GC-MS, have been essential in identifying the flavor compounds and bioactive molecules (e.g., surfactin) that characterize products like Indonesian *terasi* [13] and Malaysian *cincalok* [27]. Recent integrative studies on Japanese *shiokara* have further demonstrated how combining genomics and metabolomics provides a dual perspective on how bacterial succession directly drives metabolite shifts during processing [14]. By mapping these successions, researchers can define the chemical boundaries between high-quality maturation and the onset of spoilage, establishing a baseline for predictive quality control.

Table 1 summarizes the major microbial groups, representative taxa, functional roles, and safety-related constituents associated with high-salinity fermented seafood.

## 3. Genomic Landscapes: Decoding Microbial Potential, Safety Risks and Adaptive Traits

Genomics provides a comprehensive blueprint of the genetic potential within a microbial community, offering high-resolution insights into both food safety and quality [33]. In high-salt fermentation systems, the microbial landscape is characterized by specialized halotolerant and halophilic genera, such as *Tetragenococcus*, *Staphylococcus*, *Bacillus*, and various halophilic archaea [11,12,13]. This genetic information is important for identifying salt-adaptation traits, amino acid metabolism, biogenic amine (BA)-associated pathways, antimicrobial compound production, and specific strain-level safety concerns [17].

### 3.1. Decoding Genomic Adaptations to Osmotic Stress

To remain metabolically active under osmotic pressure, halotolerant and halophilic microorganisms require specific genomic adaptations. These include ion transport systems, compatible solute uptake pathways, stress-response regulators, and amino acid metabolism pathways that support survival under high salinity [37]. For example, genomic studies of *Tetragenococcus halophilus* and related halotolerant bacteria have revealed pathways associated with amino acid utilization and ion homeostasis [13]. Rather than merely identifying the presence of these species, genomics allows researchers to map the specific gene clusters responsible for the accumulation of compatible solutes, which serves as a baseline for understanding the adaptive capacity of the community.

### 3.2. Metagenomic Surveillance: Identifying Hazards and AMR Determinants

Metagenomic surveillance enables strain-level and community-level assessment of biological risk in fermented seafood, including the detection of opportunistic pathogens, antimicrobial resistance determinants, and virulence-associated genes [29]. Mechanistically, these genetic signals provide insight into how microbial communities may persist, compete, or contribute to safety risks under high-salinity conditions. For example, the detection of AMR genes or virulence-associated determinants should not be interpreted only as a presence-or-absence finding, but as evidence of potential ecological reservoirs that may be maintained by salt stress, biofilm formation, substrate availability, or microbial interactions during fermentation. It also facilitates the identification of low-abundance or difficult-to-culture pathogens that traditional culture-based methods often overlook. Metagenomic analysis of traditional products like Malaysian *budu* [17] has identified potentially risk-associated taxa, including *Staphylococcus cohnii* and *Clostridium disporicum*, providing a more accurate assessment of biological hazards. Furthermore, genomics serves as the primary tool for the surveillance of AMR genes, allowing researchers to monitor the occurrence and potential dissemination of resistance determinants within fermented production systems. By utilizing databases like the Comprehensive Antibiotic Resistance Database (CARD) [38], researchers can identify how specific microbial constituents may act as reservoirs for resistance.

### 3.3. Functional Genome Mining for Natural Biopreservatives

Beyond hazard identification, functional genomic mining provides a mechanistic view of how microbial communities compete, stabilize, and generate safety-relevant traits during fermentation. Genome annotation and biosynthetic gene cluster analysis are therefore most informative when interpreted in relation to biological function rather than as tool-based outputs alone. For example, the detection of bacteriocin-associated genes may indicate microbial antagonism against spoilage organisms or pathogens, while secondary metabolite pathways may suggest mechanisms of biopreservation, ecological dominance, or substrate competition under high-salt conditions.

Rapid annotation tools such as Prokka [39] can provide a functional overview of bacterial genomes, whereas specialized platforms such as BAGEL4 [40] and antiSMASH [41] support the identification of bacteriocins, ribosomally synthesized and post-translationally modified peptides, and biosynthetic gene clusters (BGCs). These predicted peptides can be compared with known bacteriocins using BACTIBASE [42], followed by experimental validation to confirm antimicrobial activity. Thus, genome mining should be viewed not simply as a cataloguing exercise, but as a hypothesis-generating approach for linking microbial genetic potential to fermentation stability, biopreservation, and safety outcomes. Representative genomic and metagenomic tools are provided in Table S1.

Thus, genomics provides the first mechanistic layer by defining the functional potential of the fermentation microbiome; however, transcriptomic analysis is required to determine which of these genetic capabilities are actively expressed under high-salinity stress.

## 4. Transcriptional Dynamics: Mechanisms of Microbial Survival Under Osmotic Stress

Transcriptomics provides a high-resolution view of microbial behavior by quantifying gene expression in response to shifting environmental conditions. This distinction is important because taxa detected by metagenomics may not be metabolically active during all fermentation stages. Currently, RNA sequencing (RNA-Seq) is the primary method for identifying how a microbial community allocates its metabolic activity during fermentation [43]. This approach is particularly effective for revealing how microorganisms adapt to the environmental filters prevalent in dry-salted foods, such as extreme salinity [44], low water activity [45], and pH fluctuations [46].

### 4.1. Quantifying the Adaptive Response to Environmental Filters

In high-salt environments, transcriptomic profiling provides mechanistic evidence of how microorganisms actively reorganize their metabolism in response to osmotic pressure. For instance, in halotolerant species like *Chromohalobacter salexigens*, salt stress triggers the expression of the *opu* family genes and *betP*, which facilitate the uptake of compatible solutes like glycine betaine to prevent cellular dehydration [44]. These expression shifts do more than ensure survival; they indicate how environmental stress can redirect microbial metabolism toward amino acid utilization, organic acid production, volatile compound formation, and stress-associated pathways that influence both safety and sensory quality.

While studies on LAB during kimchi fermentation utilize a vegetable model [47], they illustrate how transcriptomics can reveal acid-stress adaptation. This occurs through the activation of the glutamate decarboxylase (*gad*) system and arginine deiminase (*arc*) pathways, which are essential for maintaining intracellular pH homeostasis in acidic environments [48]. Similarly, in dry-cured fish, transcriptomic analysis has linked the expression of microbial lipases and proteases to the production of short-chain fatty acids and free amino acids, which serve as precursors for the product’s characteristic aroma [49].

### 4.2. Survival Strategies and Pathogen Persistence

Beyond characterizing beneficial microbes, transcriptomics is a critical tool for understanding the survival strategies that allow pathogens to persist in hostile matrices. By monitoring gene expression, researchers can identify how pathogens like *Listeria monocytogenes* activate stress-response pathways to survive salt, acid and cold stresses during food-processing [34]. Recent studies on *Salmonella* have utilized transcriptomics to map the regulatory networks involved in biofilm formation, offering a mechanistic view of how these cells may persist on processing surfaces [35]. This functional data, when integrated into the broader multi-omics framework, allows for a more comprehensive assessment of biological risks than genomic data alone [36].

### 4.3. Moving from Descriptive to Functional Synthesis

By integrating these transcriptional shifts into a broader systems biology framework, researchers can move from simple detection toward predicting community responses to processing interventions. Transcriptomics allows for the study of inter-species interactions within mixed-culture fermentations [50].

In high-salinity seafood fermentation, these interactions can determine whether amino acids are routed toward desirable flavor compounds or hazardous biogenic amines. Metatranscriptomic analysis can reveal whether microbial communities activate pathways for proteolysis, amino acid decarboxylation, acid tolerance, compatible-solute transport, or antimicrobial production under specific processing conditions. This functional information helps explain why similar microbial taxa may produce different safety and quality outcomes across fermentation batches. By identifying which pathways are active, transcriptomics provides a mechanistic basis for predicting microbial succession, substrate competition, metabolic cross-feeding, spoilage inhibition, and biogenic amine risk [51,52]. Representative transcriptomic analysis tools are listed in Table S2.

Therefore, transcriptomics bridges microbial potential and functional activity by identifying stress-responsive genes, but proteomic analysis is needed to determine whether these transcripts are translated into active enzymes and proteins that directly influence fermentation outcomes.

## 5. Functional Proteomics: Profiling Enzymatic Activity and Safety Biomarkers

While genomics and transcriptomics reveal the genetic potential and transcriptional activity of a microbial community, proteomics provides a direct assessment of the functional landscape by identifying and quantifying the proteins actively driving the fermentation process [53]. This is particularly important in fermented seafood, as gene presence and transcript levels do not always correlate with enzyme abundance or biochemical activity. The development of high-resolution liquid chromatography-tandem mass spectrometry (LC-MS/MS) has enabled comprehensive profiling of microbial proteomes, uncovering the molecular mechanisms that underpin both food safety and quality [54].

### 5.1. Enzymatic Activity and Protein-Level Salt Adaptation

In the context of dry-salted foods, proteomics has been instrumental in characterizing how halotolerant bacteria survive extreme salinity. Research into extreme halophiles, such as *Halobacterium* NRC-1, and halotolerant species like *Tistlia consotensis*, has highlighted the crucial role of general stress proteins and acidic chaperones [55,56].

Specifically, the upregulation of chaperones like DnaK and GroEL facilitates proper protein folding under high ionic strength, ensuring that essential metabolic enzymes remain functional despite osmotic pressure [57]. This protein-level adaptation is a primary driver of microbial persistence in high-brine environments, as the mere presence of stress-response genes (genomics) does not always guarantee the presence of functional, correctly folded proteins. While these models are not all derived from fermented seafood, they illustrate the protein-level mechanisms likely employed by microbes exposed to high ionic strength in salted food matrices.

### 5.2. Discovery of Functional Safety and Quality Biomarkers

Quantitative proteomics also facilitates the discovery of biomarkers that track fermentation progress and identify contamination risks [30]. In dairy and fish fermentations, proteomic studies have identified bioactive peptides with potential antimicrobial activity that inhibit the growth of spoilage organisms and pathogens, effectively extending shelf life [58].

Furthermore, proteomics supports allergen-related assessment by monitoring processing-induced changes in fish or shellfish proteins. By identifying and monitoring the abundance of allergenic proteins during food processing, this approach supports the development of safer products for sensitive consumers, aligning with international regulatory standards for food safety [59]. A notable application of proteomic technology was demonstrated in the fermentation of common carp (*Cyprinus carpio*) using *Lactobacillus plantarum* as a starter culture [60]. Researchers found that microbial proteases significantly accelerate proteolysis, breaking down muscle proteins into smaller peptides and free amino acids. This activity not only enhances savory flavor and texture but also supports microbial stability when antimicrobial peptides are produced.

### 5.3. Integrated Proteogenomics: Linking Genetic Potential to Protein Function

The integration of genomic and proteomic datasets, often termed proteogenomics [61], provides insights into the complex regulatory networks governing how microbes adapt to physical and chemical shifts during fermentation. By linking protein expression directly to the metabolic output, researchers can achieve a systems-level understanding of how interspecies interactions dictate the final chemical profile.

For example, the detection of proteases and peptidases can be interpreted alongside free amino acid profiles [62], while histidine decarboxylase abundance can be linked directly to histamine accumulation [63]. This integrated approach is critical for distinguishing between biological correlation and functional causation [64]. Representative proteomics platforms are provided in Table S3.

In this way, proteomics connects gene expression to functional biochemical machinery, while metabolomics provides the next layer of evidence by showing how these enzymatic activities are reflected in measurable chemical products such as flavor compounds, spoilage markers, and biogenic amines.

## 6. Metabolomic Signatures: Mapping Chemical Indicators of Quality and Safety

Metabolomics provides a comprehensive assessment of the low-molecular-weight compounds (<1500 Da) produced by microbial communities. This approach establishes a direct link between specific microbial metabolic activities and resulting chemical constituents that define the food’s safety and sensory profile [31]. Primary analytical techniques, such as gas chromatography-mass spectrometry (GC-MS) and nuclear magnetic resonance (NMR) spectroscopy, allow for the identification of chemical signatures associated with fermentation stage, sensory quality, spoilage, and safety risk [65]. In fermented seafood, these metabolites include free amino acids, organic acids, nucleotides, volatile aroma compounds, lipid-derived compounds, and biogenic amines.

### 6.1. Mapping Umami and Aromatic Flux

Metabolomic profiling is invaluable for identifying compounds that drive consumer preference. In fermented fish sauces, for example, metabolomics has identified high concentrations of umami-enhancing compounds, specifically glutamate and various nucleotides (e.g., IMP and GMP), which are generated through microbial proteolysis and nucleic acid degradation during extended fermentation [66]. This links metabolite accumulation to both microbial and endogenous enzymatic activity, especially protease, peptidase, and nucleotidase-mediated transformations. More broadly, volatile organic compound (VOC) profiling helps connect microbial succession with aroma-related metabolites, though seafood-specific validation remains essential [67].

### 6.2. Quantitative Safety Thresholds and Biogenic Amines

Beyond quality, metabolomics is a critical tool for detecting chemical hazards [68]. In dry-cured and fermented seafood, the accumulation of biogenic amines (BAs), such as histamine, tyramine, and cadaverine, presents a significant safety risk. To transition from descriptive analysis to a predictive framework, metabolomic data should be interpreted against regulatory or guidance-based thresholds. These threshold vary by jurisdiction, fish species, product type, and sampling framework [69]. These thresholds vary by jurisdiction, fish species, product type, and sampling framework. For example, the U.S. Food and Drug Administration Compliance Policy Guide for scombrotoxin-forming fish and fishery products identifies histamine levels of 35 mg/kg or higher as evidence of decomposition-related adulteration and 200 mg/kg or higher as a level at which the product may be considered injurious to health [70]. For fermented fish sauce, the Codex Standard for Fish Sauce specifies that the product should not contain more than 40 mg histamine/100 g, equivalent to 400 mg/kg, in any sample unit tested [71]. These values provide practical safety benchmarks for interpreting histamine accumulation in high-salinity fermented seafood and fish-sauce-like products. Metabolomic monitoring enables the detection of BA precursors and the amines themselves at early stages of production. By evaluating the metabolic flux, which is the rate at which amino acids are decarboxylated by the microbiota, producers may identify elevated-risk trajectories before final product testing [28].

### 6.3. Toward Predictive Surveillance and Process Optimization

Identifying metabolic signatures associated with contamination supports the development of rapid diagnostic tools. In high-salinity seafood matrices, such signatures may include abnormal increases in histamine, tyramine, cadaverine, putrescine, ammonia, sulfur-containing compounds, or shifts in organic acid and amino acid profiles. This is particularly valuable in traditional and artisanal production environments where fermentation conditions can be highly variable [32]. By profiling the metabolic signature of high-quality, safe products, researchers can move toward a systems-level understanding that distinguishes between simple microbial correlation and metabolic causation. This shift is necessary to standardize production and improve regulatory compliance across the industry [72]. Representative metabolomics tools and databases are provided in Table S4.

Thus, metabolomics provides the chemical endpoint of microbial activity, but causation-oriented interpretation requires integration with genomic, transcriptomic, and proteomic evidence to determine whether metabolite accumulation reflects true biological function rather than simple microbial association.

## 7. Conceptual Systems Biology Framework for Deciphering Histamine Flux in *Tetragenococcus halophilus*

**Figure 2:**
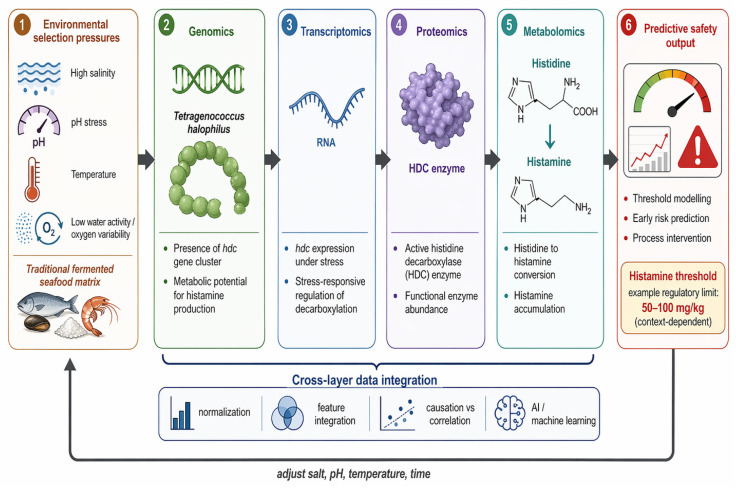
Conceptual multi-omics framework linking histidine decarboxylase activity to histamine accumulation in high-salinity fermentation. Environmental pressures such as salinity, pH, temperature, water activity, and oxygen variability shape microbial adaptation in fermented seafood. Genomics indicates the presence of the *hdc* gene cluster, transcriptomics reveals stress-responsive *hdc* expression, proteomics captures histidine decarboxylase (HDC) abundance, and metabolomics measures histidine-to-histamine conversion. Cross-layer integration supports causation-oriented interpretation, threshold modelling, and process intervention through adjustment of salt concentration, pH, temperature, and fermentation time.

To illustrate the transition from descriptive omics studies to mechanistic systems biology, histamine formation in high-salinity fermentation can be used as a conceptual model. As a major biogenic amine in fermented seafood, histamine accumulation is a critical indicator of food safety risk and can be interpreted against regulatory or guidance-based concentration thresholds. Depending on jurisdiction and product category, relevant benchmarks include 35 mg/kg as a decomposition-related action level and 200 mg/kg as a potential health-hazard level for histamine-forming fish [70], while fish sauce has a Codex maximum level of 40 mg/100 g, equivalent to 400 mg/kg [71]. In conventional studies, the mere detection of histamine-producing microorganisms or the presence of histidine decarboxylase genes is often treated as sufficient evidence of potential hazard [73]. However, these observations alone fail to explain whether histamine will actually accumulate to unsafe levels in the final product. A systems biology framework is required to connect microbial potential, functional activity, and metabolite output into a single predictive model (Figure 2).

### 7.1. From Genetic Potential to Functional Risk

At the genomic level, the identification of histamine-producing taxa, particularly halotolerant organisms such as *Tetragenococcus halophilus*, provides an initial indication of metabolic capability. Detecting the histidine decarboxylase (*hdc*) gene cluster establishes that the microbial community possesses the genetic machinery required for conversion of histidine into histamine [74]. Nevertheless, the presence of the *hdc* cluster reflects only metabolic potential, not confirmed activity. This distinction is important in traditional fermented seafood, where microbial communities are highly dynamic and environmental conditions may either suppress or stimulate amine production.

A more informative interpretation emerges when genomic information is linked to ecological context. In high-salinity fermentation systems, microbial behavior is shaped by multiple environmental filters, including salt concentration, pH, temperature, oxygen availability, and water activity [75]. These factors do not merely determine which organisms survive; they also influence whether histamine decarboxylation pathways are activated. Accordingly, genomic detection should be interpreted as the first layer of evidence within a broader systems model, rather than as a stand-alone predictor of food safety risk.

### 7.2. Transcriptional and Proteomic Regulation of Histamine Formation

Transcriptomic analysis adds a critical functional dimension by revealing whether *hdc*-associated genes are actively expressed under specific fermentation conditions. In stressful environments, decarboxylation pathways may contribute to microbial stress adaptation, particularly through pH homeostasis and survival under acidic or osmotic pressure [76]. Thus, increased *hdc* expression suggests that histamine formation is not a passive biochemical event, but part of an active physiological response. This helps explain why the presence of histamine-producing bacteria does not always correspond directly to histamine accumulation across all products or processing stages.

Proteomics extends this further by determining whether histidine decarboxylase is translated into its functional form. This layer is essential because transcription does not always translate into enzyme abundance [77]. By combining transcriptomic and proteomic evidence, the analysis moves from genetic possibility to biochemical functionality. In this context, Figure 2 highlights the progression from *hdc* gene presence to stress-responsive *hdc* expression and finally to active HDC enzyme abundance, clarifying the mechanistic basis of histamine production in complex seafood matrices.

### 7.3. Metabolomic Readout and Predictive Safety Modelling

The metabolomic layer provides the most direct evidence of safety outcomes by measuring substrate depletion and metabolite accumulation, particularly the conversion of histidine into histamine. Unlike genomic or transcriptomic signals, metabolomics captures the end result of microbial activity, linking functionality to product safety in a measurable manner [78]. When evaluated together with genomic, transcriptomic, and proteomic data, metabolomics enables a robust interpretation of whether histamine levels reflect true functional causation rather than simple correlation.

Cross-layer integration of these datasets supports the development of predictive safety frameworks. Rather than waiting for end-product testing to reveal excessive histamine, an integrated model can estimate risk trajectories during fermentation. As illustrated in Figure 2, this approach utilizes normalization, feature integration, and AI-assisted modelling to support threshold-oriented monitoring. Practically, the framework provides a basis for adjusting salinity, pH, or temperature before histamine reaches unsafe concentrations. Thus, integrated multi-omics transforms histamine assessment from a descriptive record of contamination into a predictive tool for risk management.

## 8. Technical Challenges in Multi-Omics Data Integration

The integration of genomics, transcriptomics, proteomics, and metabolomics provides a systems-level view of microbial ecosystems, but the technical execution faces significant hurdles. A primary challenge is the “curse of dimensionality,” where the high number of biological variables (e.g., thousands of transcripts) relative to a small number of samples can lead to overfitting in predictive models [79].

### 8.1. Normalization and Cross-Platform Variability

To move from descriptive observations to predictive modeling, data must be rigorously standardized. Transcriptomic counts (e.g., TPM) and proteomic intensities (e.g., LFQ) have fundamentally different distributions and scales. Without proper Z-score normalization or log-transformation, a single high-volume data layer can “mask” functionally critical but lower-volume signals from other layers [80].

Furthermore, addressing cross-platform variability is essential when merging datasets from different sequencing or mass spectrometry runs. Batch effects, arising from sample preparation, extraction chemistry, sequencing depth, instrument drift, and database choice can strongly influence cross-omics comparisons [81]. Therefore, candidate biomarkers or predictive features should be validated using independent batches, targeted assays, or controlled fermentation experiments to ensure that identified patterns reflect biological variation rather than technical artifacts.

### 8.2. Distinguishing Correlation from Causation

A common pitfall in food-omics is assuming that the presence of a gene (correlation) implies the presence of a metabolite (causation). By mapping genetic potential to functional outcomes, multi-omics frameworks offer a deeper understanding of how microbial communities influence food safety and quality [82]. Merging transcriptomic and metabolomic data can reveal “metabolic cross-talk,” where the metabolites produced by one species—such as organic acids or signaling molecules—influence the gene expression and survival of another.

To strengthen causal inference, researchers are increasingly adopting intermediate integration strategies, such as Multi-block Partial Least Squares (MB-PLS), which identify common variance across layers and provide a more robust basis for predicting microbial behavior [83].

### 8.3. AI-Driven Integration and Predictive Modeling

Advances in bioinformatics have been essential for handling the high-dimensional datasets typical of systems biology. Machine learning algorithms are now frequently used to detect non-linear patterns across different omics layers, supporting the creation of predictive models for microbial behavior [84,85]. These computational tools are especially beneficial for decision-support systems, where the rapid interpretation of microbial or metabolite data can inform process optimization and active risk management [86]. Representative multi-omics integration platforms are provided in Table S5, while Table 2 summarizes the biological contribution of each omics layer to predictive safety assessment.

## 9. Future Perspectives: From Single-Cell Resolution to Digital Traceability

The rapid evolution of omics technologies is fundamentally altering the trajectory of food safety research. While current methods provide a population-level (bulk) view of the microbiota, emerging techniques such as single-cell omics and spatial transcriptomics offer even deeper insights into the functional heterogeneity of microbial ecosystems [87].

### 9.1. High-Resolution Omics: Single-Cell and Spatial Analysis

Moving beyond bulk analysis, single-cell omics allows researchers to reveal the functional diversity of individual cells within a community [88]. In high-salinity matrices, microbes are not uniformly distributed; understanding their spatial organization—for instance, within the protein-rich layers of dry-salted fish—is essential for mechanistically modeling how these micro-environments influence both spoilage and safety. Spatial transcriptomics, in particular, can map metabolic activity directly onto the food’s physical structure, providing a geographical context to microbial succession [89].

### 9.2. Artificial Intelligence and the “Digital Twin” Concept

Artificial intelligence (AI) and machine learning will continue to play transformative roles in synthesizing high-dimensional multi-omics data [90,91]. By identifying non-linear patterns across genomic and metabolomic layers, these tools enable the development of “Digital Twins” for fermentation processes. Unlike current descriptive models, a Digital Twin is a virtual representation that simulates microbial and metabolic trajectories in real-time [92]. This technology could predict how subtle shifts in temperature or salinity might influence the final concentration of biogenic amines, allowing for proactive process interventions rather than relying solely on end-product rejection.

### 9.3. Data Standardization and Global Traceability

To realize the full potential of these innovations, robust collaboration between academia, industry stakeholders, and regulatory agencies is required [93]. Harmonized metadata reporting, validated marker panels, and agreed-upon safety benchmarks will be necessary before omics-based monitoring can be implemented in industrial settings. Establishing standardized protocols and promoting open-access data sharing is necessary to bridge the gap between laboratory research and real-world application. Furthermore, because traditional fermented foods are culturally embedded, public engagement is important; the acceptance of omics-guided monitoring will depend on trust, transparency, and clear communication regarding its safety benefits [94].

## 10. Conclusions

Omics technologies have fundamentally transformed the study of food safety by providing a mechanistic view through which to view the microbial ecosystems driving traditional fermentation. This review demonstrates that genomics, transcriptomics, proteomics, and metabolomics each offer distinct yet highly complementary perspectives on microbial diversity and the functional regulation of safety-critical constituents. When integrated, these disciplines provide a holistic understanding of how high-salinity environments select for specialized microorganisms that define the biochemical stability, quality, and shelf life of fermented seafood products.

The transition from descriptive, tool-focused research to functional, predictive modeling—facilitated by AI and advanced bioinformatics—marks a new era in food microbiology. By adopting emerging techniques such as single-cell analysis and mechanistic frameworks that distinguish between biological causation and simple correlation, the food industry can significantly enhance the consistency and safety of artisanal products.

As illustrated through the conceptual framework of histamine regulation, the integration of metabolic activity, enzyme abundance and quantitative safety thresholds enables a shift toward proactive risk management. Ultimately, refined and validated multi-omics frameworks can help build more resilient, consistent, and transparent high-salinity fermented seafood systems globally.

## Figures and Tables

**Table 1:** Major microbial groups, functional roles, and safety-related constituents in high-salinity fermented seafood.

Microbial Group	Representative Taxa	Functional Role in High-Salinity Fermentation	Key Constituents or Metabolites	Safety and Quality Relevance	References
Halotolerant lactic acid bacteria (LAB)	*Tetragenococcus halophilus*, *Lactobacillus* spp.	Acid tolerance, amino acid metabolism, microbial stabilization	Organic acids, amino acid derivatives, bacteriocin-like compounds, biogenic amines	Contributes to flavor development and microbial stability, but some strains may participate in histamine formation	[7,8,11,14,23,26,28]
Coagulase-negative staphylococci (CNS)	*Staphylococcus* spp.	Proteolysis, lipolysis, aroma development	Peptides, free amino acids, aldehydes, alcohols, esters	Supports sensory maturation, but strain-level safety assessment is needed because some species may carry virulence or resistance traits	[9,12,17,29]
*Bacillus* species	*Bacillus subtilis*, *Bacillus* spp.	Enzyme production, peptide release, substrate degradation	Proteases, lipases, bioactive peptides, surfactin-like compounds	May contribute to flavor and biopreservation, but uncontrolled growth may cause spoilage or safety concerns	[13,27,30]
Halophilic archaea	*Halobacterium*, *Halococcus* spp.	Adaptation to extreme salt, pigment production, late-stage succession	Carotenoids, compatible solutes, volatile compounds	May influence color, aroma, and late-stage microbial ecology in highly salted matrices	[9,12]
Spoilage-associated bacteria	*Clostridium*, *Pseudomonas*, some enterobacteria	Protein degradation, off-odor formation, toxin or amine production	Cadaverine, putrescine, ammonia, sulfur compounds	Associated with spoilage, undesirable sensory changes, and safety risk	[10,17,28,31,32]
Foodborne pathogens or opportunistic contaminants	*Listeria monocytogenes*, *Salmonella* spp., pathogenic *Staphylococcus* spp.	Stress survival, biofilm formation, persistence under processing conditions	Virulence markers, toxins, antimicrobial resistance determinants	Important targets for surveillance, risk assessment, and process control	[10,29,33,34,35,36]

**Table 2:** Mechanistic contribution of each omics layer to predictive safety assessment in high-salinity fermentation.

Omics Layer	Biological Question	Mechanistic Readout	Example in High-Salinity Fermentation	Predictive Value	References
Genomics/Metagenomics	Which organisms and genes are present?	Taxonomic composition, *hdc* genes, AMR genes, bacteriocin gene clusters	Detection of *Tetragenococcus halophilus*, *Staphylococcus* spp., AMR determinants, or histidine decarboxylase genes	Identifies microbial potential and possible safety hazards	[11,17,29,33,38,39,40,41,42]
Transcriptomics/Metatranscriptomics	Which genes are actively expressed under stress?	Expression of osmotic stress genes, acid-resistance genes, decarboxylase genes, biofilm-related genes	Upregulation of compatible solute transporters or *hdc* expression under salt and pH stress	Indicates active microbial response rather than passive gene presence	[34,35,36,43,44,45,46,48,49,50,51,52]
Proteomics/Metaproteomics	Which enzymes and proteins are functionally active?	Abundance of proteases, lipases, chaperones, histidine decarboxylase, antimicrobial peptides	Detection of stress proteins, active HDC enzyme, or proteolytic enzymes during fermentation	Links gene expression to functional biochemical activity	[30,53,54,55,56,59,60,61,64]
Metabolomics	What chemical products accumulate?	Amino acids, organic acids, VOCs, histamine, tyramine, cadaverine	Monitoring histidine depletion, histamine accumulation, and aroma-related metabolites	Provides direct evidence of safety, spoilage, and sensory outcome	[13,14,28,31,32,65,66,67,72]
Integrated Multi-Omics	How do molecular layers interact?	Cross-layer associations, causal networks, flux models, predictive risk trajectories	Linking *hdc* gene presence, *hdc* expression, HDC abundance, and histamine accumulation	Supports early warning systems and process intervention	[64,82,84,85,86]

## Data Availability

Data sharing is not applicable to this article as no datasets were generated or analyzed during the current study.

## Supplementary Materials

The following supporting information can be downloaded at https://www.mdpi.com/article/10.3390/biology15100772/s1. Supplementary File S1: Bioinformatics and analytical tools for multi-omics analysis in high-salinity fermented seafood. This file includes Table S1, Bioinformatics tools for genomic and metagenomic analysis. The selected tools focus on bridging raw sequence data to the biological mechanisms of halotolerance and pathogen surveillance in fermented food research [38,39,40,41,95,96,97,98,99,100,101]; Table S2, Essential transcriptomics tools for microbial gene expression and pathway analysis. These platforms are utilized for decoding microbial adaptive signatures and quantifying transcriptional shifts under high-salinity environmental filters [102,103,104,105,106,107,108,109,110,111]; Table S3, Key proteomics tools for functional profiling and safety assessment. This suite of tools is used for profiling functional enzymatic shifts and identifying microbial protein biomarkers within high-brine food matrices [112,113,114,115,116,117,118,119,120,121]; Table S4, Essential metabolomics tools and databases for food safety and quality profiling. These resources support the quantitative safety screening and the mapping of chemical constituents that define the sensory and toxicological profiles of fermented seafood [122,123,124,125,126,127,128,129,130,131]; and Table S5, Summary of advanced bioinformatics tools and platforms for multi-omics data integration. The listed frameworks emphasize predictive modeling and the mechanistic synthesis of microbial functionality, moving beyond descriptive observations to address technical hurdles such as data normalization and dimensionality [132,133,134,135,136,137,138,139,140,141].
