# Supplementary material for: Mechanistic Systems Biology of High-Salinity Fermented Seafood: Multi-Omics Integration for Microbial Safety and Quality Prediction
Source: Biology (Basel). 2026 May 12;15(10):772. doi: 10.3390/biology15100772 (PMC13203410; doi:10.3390/biology15100772)
Supplement: Supplementary file 1 [file biology-15-00772-s001.zip › biology-4316833-supplementary.pdf]

Table S1. **Bioinformatics tools for genomic and metagenomic analysis.** The selected tools focus on bridging raw sequence data to the biological mechanisms of halotolerance and pathogen surveillance in fermented food research.

Tool	Primary Function	Application in Food Safety	Analytical Output	Reference
SPAdes	Genome Assembly	High-quality *de novo* assembly of microbial genomes	Contig and scaffold sequences (.fasta)	[95]
QUAST	Quality Assessment	Verifying the accuracy of genome assemblies	N50 metrics and misassembly reports	[96]
Prokka	Rapid Genome Annotation	Identifying functional genes in foodborne pathogens	Annotated .gff and .gbk files	[39]
antiSMASH	BGC Identification	Mining genomes for novel bacteriocins and metabolites	Gene cluster maps and chemical structures	[41]
BAGEL4	Bacteriocin Mining	Detecting ribosomally synthesized antimicrobial peptides	Predicted peptide sequences and motifs	[40]
MEGAHIT	Metagenomic Assembly	Analyzing complex mixed-cultures in fermented foods	Reconstructed metagenomes from raw reads	[97]
CARD	AMR Gene Prediction	Detecting antimicrobial resistance (AMR) genes in food	Resistance gene heatmaps and ontology	[38]
PathogenWatch	Pathogen Surveillance	Tracking and identifying the source of foodborne pathogens	Phylogenetic trees and resistance profiles	[98]
PubMLST	Strain Typing	Tracing contamination origins in food outbreaks	Sequence types (ST) and clonal complexes	[99]
Roary	Pangenome Analysis	Comparing multiple microbial strains for unique virulence genes	Core vs. accessory gene distribution	[100]
Panseq	Accessory Genome Detection	Differentiating pathogenic strains from safe commensals	Unique genomic regions and SNP analysis	[101]

Table S2: **Essential transcriptomics tools for microbial gene expression and pathway analysis.** These platforms are utilized for decoding microbial adaptive signatures and quantifying transcriptional shifts under high-salinity environmental filters.

Tool	Primary Function	Application in Food Safety	Analytical Advantage	Reference
DESeq2	Differential Expression	Detecting genes upregulated during salt or acid stress	Robust statistical handling of small sample sizes	[102]
Salmon	Transcript Quantification	Rapidly measuring gene expression levels in large datasets	Alignment-free; extremely high computational speed	[103]
Rockhopper	Bacterial RNA-Seq Analysis	Mapping operons and non-coding RNAs in food microbes	Specifically optimized for prokaryotic transcriptomes	[104]
Cufflinks	Transcript Assembly	Monitoring transcript isoforms during fermentation phases	Accurate assembly of transcripts without a reference	[105]
iDEP	Pathway Enrichment	Identifying metabolic pathways affected by food processing	Integrated web-interface for rapid visualization	[106]
STRING-db	Network Analysis	Mapping functional interactions between microbial proteins	Visualizes protein-protein interaction networks	[107]
RSEM	Expression Quantification	Estimating gene and isoform abundances	High accuracy in multi-mapping read handling	[108]
Ballgown	Statistical Analysis	Investigating virulence-related gene expression	Seamless integration with transcript assembly tools	[109]
TopHat	Read Alignment	Identifying splice sites and stress-response markers	Reliable alignment of reads to complex genomes	[110]
Geneious	Multi-purpose Platform	Visualizing microbial adaptation to processing environments	User-friendly GUI for end-to-end analysis	[111]

Table S3. Key proteomics tools for functional profiling and safety assessment. This suite of tools is used for profiling functional enzymatic shifts and identifying microbial protein biomarkers within high-brine food matrices.

Tool	Description	Application in Food Safety	Primary Analytical Benefit	Reference
MASCOT	Protein search engine	Identifying allergens in processed foods	Industry-standard for peptide mass fingerprinting	[112]
MaxQuant	MS data analysis	Quantifying antimicrobial proteins	High-accuracy label-free quantification (LFQ)	[113]
Proteome Discoverer	LC-MS/MS platform	Characterizing stress-response proteins	Comprehensive workflow for post-translational modifications	[114]
PeptideAtlas	Peptide repository	Monitoring bioactive peptides	Large-scale validation of identified protein sequences	[115]
SWATH-MS	Data-independent acquisition	Identifying contamination biomarkers	High reproducibility and data completeness across samples	[116]
X! Tandem	Peptide identification	Studying proteomes in complex matrices	Open-source and highly customizable search parameters	[117]
Scaffold	Visualization tool	Analyzing protein interaction networks	Statistical validation and comparison of multiple datasets	[118]
OpenMS	Analysis framework	Monitoring changes during fermentation	Flexible, modular architecture for custom omics workflows	[119]
Skyline	Targeted proteomics	Quantifying specific toxins or allergens	Highly precise quantification of pre-selected target proteins	[120]
FragPipe	Analysis pipeline	Protein quantification in mixed cultures	Extremely fast search speeds for large-scale datasets	[121]

Table S4. Essential metabolomics tools and databases for food safety and quality profiling. These resources support the quantitative safety screening and the mapping of chemical constituents that define the sensory and toxicological profiles of fermented seafood.

Tool	Primary Function	Application in Food Safety	Analytical Benefit	Reference
MetaboAnalyst	Statistical Analysis	Identifying spoilage markers in fermented foods	Comprehensive platform for multivariate data (PCA/PLS-DA)	[122]
XCMS Online	Data Processing	Profiling metabolic markers of foodborne pathogens	Cloud-based untargeted metabolomics workflow	[123]
MZmine	Peak Detection	Monitoring volatile flavor compounds	Open-source tool for mass spectrometry data visualization	[124]
LIMSA	Amine/Lipid Analysis	Detecting toxic biogenic amines in salted foods	Specialized for quantitative analysis of complex small molecules	[125]
BAYESIL	NMR Profiling	Analyzing metabolites in traditional products	Fully automated identification and quantification of NMR spectra	[126]
GC-MS	Volatile Profiling	Identifying volatile organic compounds (VOCs)	High-resolution detection of aroma-active compounds	[127]
MetFrag	Compound Annotation	Detecting unknown metabolites in microbial niches	Fragmentation-based identification of "dark" metabolites	[128]
MassBank	Structure Validation	Validating metabolite identities in safety studies	High-quality mass spectral database for structural confirmation	[129]
NMRShiftDB	NMR Database	Identifying spoilage-related compounds	Open-access repository for NMR chemical shift data	[130]
Compound Discoverer	Metabolite Identification	Mapping metabolic shifts during fermentation	Advanced software for unknown identification and pathway mapping	[131]

Table S5: **Summary of advanced bioinformatics tools and platforms for multi-omics data integration.** The listed frameworks emphasize predictive modeling and the mechanistic synthesis of microbial functionality, moving beyond descriptive observations to address technical hurdles such as data normalization and dimensionality.

Tool	Primary Function	Application in Food Safety	Integration Strategy	Reference
Omics Pipe	Automated Pipeline	Linking genetic and protein data for safety	Orchestrates multi-step workflows for NGS and MS data	[132]
Metascape	Pathway Analysis	Studying microbial adaptation to processing	Consolidates functional annotations across omics layers	[133]
Cytoscape	Network Visualization	Mapping interactions in mixed cultures	Visualizes complex interspecies and protein networks	[134]
KBase	Predictive Modeling	Modeling microbial food ecosystems	Cloud-based platform for community metabolic modeling	[135]
QIIME2	Microbiome Analysis	Studying ecosystems in fermented foods	Integrates taxonomic data with metabolic potential	[136]
Phyloseq	Data Integration	Linking metagenomics and metabolomics	R-based package for high-throughput phylogenetic data	[137]
Galaxy-P	Multi-omics Platform	Supporting workflows for food microbes	Specialized for proteogenomics and metabolomics	[138]
ML-based Integration	Machine Learning	Predicting contamination risks in real-time	Uses deep learning to correlate multi-layer data patterns	[139]
iCluster	Integrative Clustering	Grouping data to study microbial interactions	Joint latent variable modeling for different data types	[140]
MiBiOmics	Interactive Exploration	Analyzing the interplay of microbes and safety	Web-based exploration of correlations between layers	[141]
